# Efficacy and safety of robot-assisted laparoscopic myomectomy versus laparoscopic myomectomy: a systematic evaluation and meta-analysis

**DOI:** 10.1186/s12957-023-03104-8

**Published:** 2023-07-28

**Authors:** Yannan Sheng, Ziqiang Hong, Jian Wang, Baohong Mao, Zhenzhen Wu, Yunjiu Gou, Jing Zhao, Qing Liu

**Affiliations:** 1 The First Clinical Medical College, Gansu University of Chinese Medicine, Lanzhou, China; 2 Obstetrics and Gynecology, Gansu Maternal and Child Health Hospital, Lanzhou, China; 3 Thoracic Surgery Center, Gansu Provincial People’s Hospital, Lanzhou, China; 4 Lanzhou First People’s Hospital, Lanzhou, China

**Keywords:** Robotics, Laparoscopes, Uterine fibroids, Myomectomy, Meta-analysis

## Abstract

**Objective:**

Systematic evaluation of the efficacy and safety of robotic-assisted laparoscopic myomectomy (RALM) versus laparoscopic myomectomy (LM).

**Methods:**

PubMed, Embase, The Cochrane Library, and Web of Science database were searched by computer to seek relevant literature in order to compare the efficacy and safety of RALM with that of LM from the establishment of the databases to January 2023, and Review Manager 5.4 software was utilized to perform a meta-analysis on the literature.

**Results:**

A total of 15 retrospective clinical controlled studies were included. There exists a total of 45,702 patients, among 11,618 patients in the RALM group and the remaining 34,084 patients in the LM group. Meta-analysis results revealed that RALM was associated with lesser intraoperative bleeding (MD =  − 32.03, 95%CI − 57.24 to − 6.83, *P* = 0.01), lower incidence of blood transfusions (OR = 0.86, 95%CI 0.77 to 0.97, *P* = 0.01), shorter postoperative hospital stay (MD =  − 0.11, 95%CI − 0.21 to − 0.01, *P* = 0.03), fewer transitions to open stomach (OR = 0.82, 95%CI 0.73 to 0.92, *P* = 0.0006), and lower incidence of postoperative complications (OR = 0.58, 95%CI 0.40 to 0.86, *P* = 0.006) than LM, whereas LM is more advantageous in terms of operative time (MD = 38.61, 95%CI 19.36 to 57.86, *P* < 0.0001). There was no statistical difference between the two surgical methods in terms of maximum myoma diameter (MD = 0.26, 95%CI − 0.17 to 0.70, *P* = 0.24).

**Conclusion:**

In the aspects of intraoperative bleeding, lower incidence of blood transfusions, postoperative hospital stay, transit open stomach rate, and postoperative complications, RALM has a unique advantage than that of LM, while LM has advantages over RALM in terms of operative time.

## Introduction

Uterine fibroids are common among women, with a prevalence of 20 to 40% among women of childbearing age. Patients may have no obvious symptoms or may suffer from increased menstrual flow, anemia, urinary frequency, urinary urgency, and other discomforts [1]. In clinical practice, surgical treatment is often performed on patients who meet the indications based on the type, size, and number of fibroids. At present, the main procedures used are myomectomy and hysterectomy, etc. Myomectomy can preserve the integrity of the patient’s reproductive organs and fertility to the greatest extent and is the preferred procedure for patients with uterine fibroids [2]. Traditional open myomectomy is effective but more invasive. In recent years, with the advancements in laparoscopic surgery and the introduction of da Vinci robotic-assisted laparoscopy, gynecologists have gained new surgical options for performing uterine fibroid resection. Robotic-assisted laparoscopic myomectomy has the advantages of clear three-dimensional vision, precise and flexible operation, and easy-to-master surgical technique [3]. Several studies [4, 5] have confirmed the feasibility and safety of robot-assisted or laparoscopic treatment of uterine fibroids. However, these studies were single-center retrospective studies with limited sample sizes. The jury is still out on whether the robot can achieve the same or even better surgical results than laparoscopy in the treatment of uterine fibroids. Although a meta-analysis [6] from 5 years ago compared the efficacy of the two, the number of included papers was small and robotic surgery underwent rapid development in the last 5 years. Therefore, more recent literature was included in this study and a more comprehensive meta-analysis was conducted with a view to providing a higher level of evidence-based medical proof for clinical practice.

## Materials and methods

This meta-analysis was performed by the Preferred Reporting Items for Systematic Reviews and Meta-Analyses (PRISMA) guidelines and was registered in PROSPERO (CRD42022324807).

### Literature search methods

Computer searches of PubMed, EMbase, The Cochrane Library, and Web of Science using a combination of subject terms plus free terms. Search the published literature comparing the efficacy of robotic-assisted myomectomy with laparoscopic myomectomy for the period built to January 2023. English search terms: robotic surgical procedures, robotic surgery, robot-assisted surgery, da Vinci, laparoscopes, laparoscopy, uterine fibroids, and myomectomy. Using PubMed as an example, the specific searches are as follows: (((“Robotic Surgical Procedures” [Mesh]) or ((((robotic surgery [Title/Abstract]) or (robot assisted surgery [Title/Abstract])) or (da Vinci [Title/Abstract])) or (Da Vinci [Title/Abstract]))) and ((“Laparoscopy” [Mesh]) or ((((laparoscope [Title/Abstract]) Or (laparoscopic surgery [Title/Abstract])) or (celioscope [Title/Abstract])) or (peritoneoscopes [Title/Abstract])))) and (((uterine fibroids [Title/Abstract]) or (hysteromyoma [Title/Abstract])) or (myomectomy [Title/Abstract])).

### Eligibility criteria

Inclusion criteria: (i) type of study to be included: a reasonably designed retrospective study or a randomized controlled trial, whether or not blinded, provided that the two data sets are controlled; (ii) study population: patients diagnosed with uterine fibroids and undergoing myomectomy; (iii) interventions: robot-assisted myomectomy versus laparoscopic myomectomy, with a detailed description of both procedures; and (iv) outcome indicators: operative time, intraoperative bleeding, incidence of blood transfusions, length of hospital stay, rate of intermediate openings, rate of postoperative complications, and maximum myoma diameter.

Exclusion criteria: (i) non-clinically controlled studies such as reviews, case reports, empirical summaries, or single-arm efficacy observations; (ii) non-English literature (Chinese literature), poor quality literature; (iii) no useful data could be extracted or the full text was not available.

### Literature screening and data extraction

Two gynecologists independently screen the literature, extract the data, and then cross-check, and if disagreements arise, they are resolved by a third gynecologist’s decision or through group discussion. The following data were extracted for each study: (i) first author’s name, year of publication, study start date, country, article type, patient group and number, and age and body mass index (BMI); (ii) outcome indicators of interest: operative time, intraoperative bleeding, incidence of blood transfusions, length of hospital stay, rate of intermediate openings, rate of postoperative complications, and maximum myoma diameter.

### Quality evaluation of the included studies

The quality of the included cohort studies was evaluated using The Newcastle–Ottawa Scale (NOS), which consists of 8 entries with a total score of 9 [7].

### Statistical analysis

We will use the Review Manager (version 5.4) software provided by the Cochrane Collaboration Network to perform meta-analysis on the data from the included studies. The effect indicators are as follows: odds ratio (OR) for dichotomous information and mean difference (MD) for continuous variables. All effect sizes are expressed as a 95% confidence interval (CI); 95% CI and OR or MD are calculated for each study effect indicator, and heterogeneity should be tested before combining effect sizes. Heterogeneity between the results of the studies included in this meta-analysis was calculated using the default *Q* test of Review Manager (version 5.4) software to calculate chi^2^, *I*^2^. If *I*^2^ < 50% and *P* > 0.1, heterogeneity between studies was considered insignificant and the data were analyzed using a fixed effects model; if *I*^2^ > 50% and *P* ≤ 0.1, heterogeneity between studies was considered significant and the data were analyzed using a random effects model. In addition, publication bias in this study was analyzed using funnel plots.

## Results

### Literature search results

**Fig. 1:**
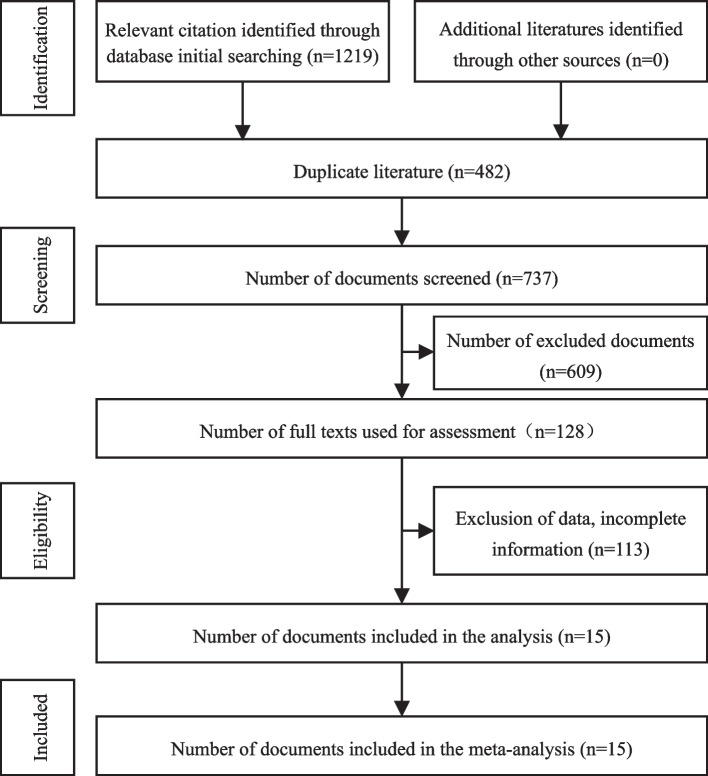
Flow diagram of literature retrieval and screening

A total of 1219 articles were obtained, and after eliminating duplicates by Endnote X9, and then eliminating irrelevant papers by reading the title and abstract, 15 articles were retained after reading the full text [8–22]. See Table 1 for basic information on the selected literature. A total of 45,702 patients, 11,618 patients in the RALM group and 34,084 patients in the LM group. The literature screening process and results are shown in Fig. 1.

**Table 1:** Basic information about the included studies

First author, year	Study date	Country	Group	Patients	Age (years)	BMI (kg/m^2^)	NOS score
Bedient 2009 [8]	2000–2008	USA	RALM	41	43.00 ± 12.00	24.7 ± 5.0	8
			LM	40	40.90 ± 6.60	25.3 ± 5.4	
Nezhat 2009 [9]	2006–2007	USA	RALM	15	39.00 ± 5.50	23 (18–31)	8
			LM	35	41.00 ± 6.75	24 (19–33)	
Barakat 2011 [10]	1995–2009	USA	RALM	89	37.00 ± 1.75	25.15 (22.14–29.44)	7
			LM	93	38.00 ± 2.25	24.10 (22.00–28.01)	
Gargiulo 2012 [11]	200–2009	USA	RALM	174	38.00 ± 8.75	25.1 (18.4–53)	8
			LM	115	39.00 ± 7.25	25.1 (17.5–54.9)	
Hsiao 2013 [12]	2010–2011	Taiwan	RALM	20	47.00 ± 1.25	24.2 (22.9–25.6)	8
			LM	22	48.00 ± 1.00	23.1 (21.9–25.1)	
Ahmet 2013 [13]	2008–2010	Turkey	RALM	15	34.20 ± 5.65	25.64 ± 3.29	7
			LM	23	35.70 ± 6.13	27.60 ± 5.18	
Gobern 2013 [14]	2007–2009	USA	RALM	66	40.00 ± 6.25	25 (19–52)	7
			LM	73	39.00 ± 8.25	27 (19–53)	
Pluchino 2014 [15]	1999–2001	Italy	RALM	70	34.72 ± 5.95	22.86 (17–35)	8
			LM	69	36.40 ± 7.14	23.84 (20.5–28.5)	
Ngan 2017 [16]	2008–2012	USA	RALM	10,677	NA	NA	8
			LM	33,088	NA	NA	
MacKoul 2018 [17]	2011–2013	USA	RALM	156	36.5 ± 5.7	28.6 ± .7	8
			LM	163	37.1 ± 7.3	27.7 ± 6.5	
Takmaz 2018 [18]	2016–2017	Turkey	RALM	31	38 ± 5	23 ± 4	7
			LM	33	35 ± 5	24 ± 4	
Chen 2018 [19]	2012–2016	Taiwan	RALM	26	41 (39–46)	23.7 (20.7–26.5)	8
			LM	52	47 (44–49)	25.2 (22.1–28.6)	
Sheu 2019 [20]	2014–2017	Taiwan	RALM	93	39 ± 6.7	21.9 ± 2.9	7
			LM	110	39 ± 6.1	22.4 ± 3.5	
Won 2020 [21]	2017–2019	Korea	RALM	121	39.1 ± 5.8	22.7 ± 3.0	8
			LM	144	39.3 ± 5.6	22.9 ± 4.1	
Morales 2022 [22]	2010–2018	Mexico	RALM	24	35.23 ± 4.19	23.38 ± 1.77	7
			LM	24	37.24 ± 5.65	24.62 ± 3.28	

*Abbreviations*: *BMI* body mass index, *NA* no relevant data available

### Quality evaluation of the included literature

The NOS scores for the included cohort studies are shown in Table 1, and all studies were of high quality with NOS scores between 7 and 9.

### Meta-analysis results

#### Comparison of operation times

**Fig. 2:**
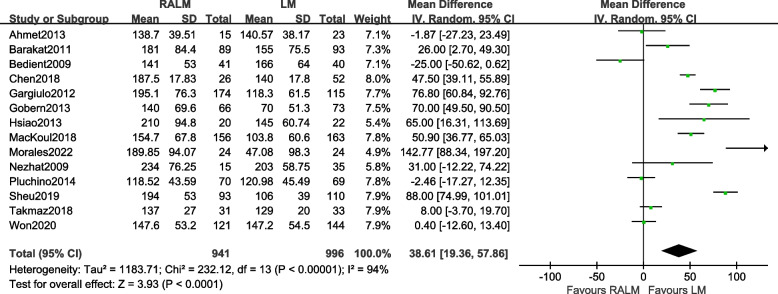
Meta-analysis forest plot for operative time

A total of 14 studies were included [8–15, 17–22]. The results [MD = 38.61, 95%CI (19.36, 57.86), *P* < 0.0001] indicate a statistically significant difference between the two surgical approaches in terms of operative time, suggesting that the LM group has shorter operative time than the RALM group, the results of the meta-analysis are shown in Fig. 2.

#### Comparison of intraoperative bleeding

**Fig. 3:**
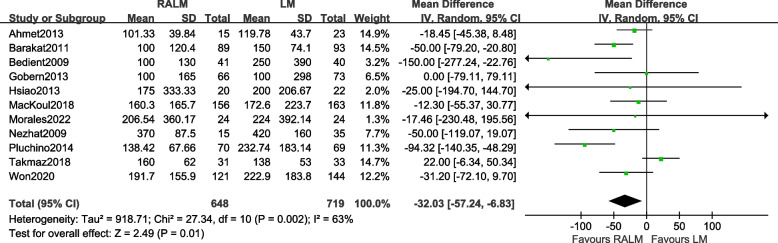
Meta-analysis forest plot of intraoperative bleeding

A total of 11 studies [8–10, 12–15, 17, 18, 21, 22]. The results [MD =  − 24.67, 95% CI (− 41.91, − 7.43), *P* = 0.005] demonstrate a statistically significant difference in intraoperative bleeding between the two surgical approaches. This suggests that the RALM group exhibits lower levels of intraoperative bleeding compared to the LM group. The findings of the meta-analysis are presented in Fig. 3.

#### Comparison of the incidence of blood transfusions

**Fig. 4:**
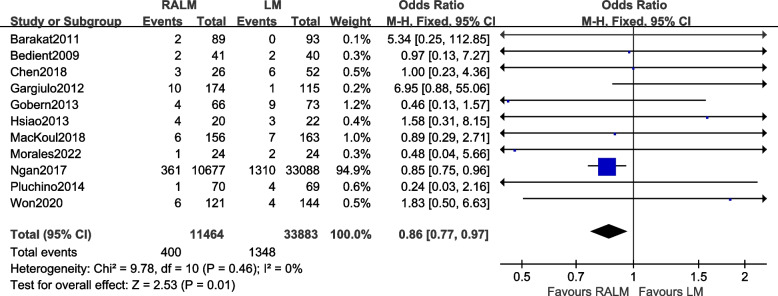
Meta-analysis of the forest for the incidence of blood transfusions

A total of 11 studies were included [8, 10–12, 14–17, 19, 21, 22]. The results [OR = 0.86, 95% CI (0.77, 0.97), *P* = 0.01] reveal a statistically significant difference in the incidence of blood transfusions between the two procedures. This implies that the RALM group had a lower rate of blood transfusion compared to the LM group. The meta-analysis findings are presented in Fig. 4.

#### Comparison of hospital stay

**Fig. 5:**
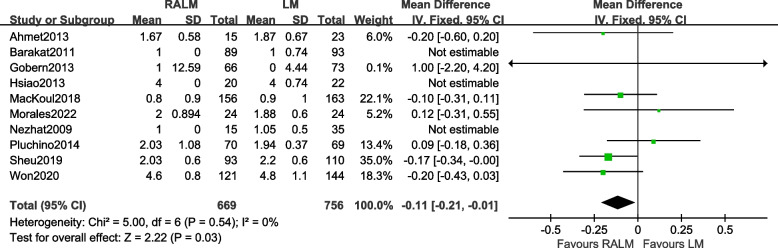
Meta-analysis forest plot for hospital stay

A total of 10 studies were included [9, 10, 12–15, 17, 20–22]. The results [OR =  − 0.11, 95%CI (− 0.21, − 0.01), *P* = 0.03] illustrate a statistically significant difference in the length of stay between the two surgical procedures, indicating that the RALM group has a lesser length of stay than the LM group; the results of the meta-analysis are shown in Fig. 5.

#### Comparison of transit open belly rate

**Fig. 6:**
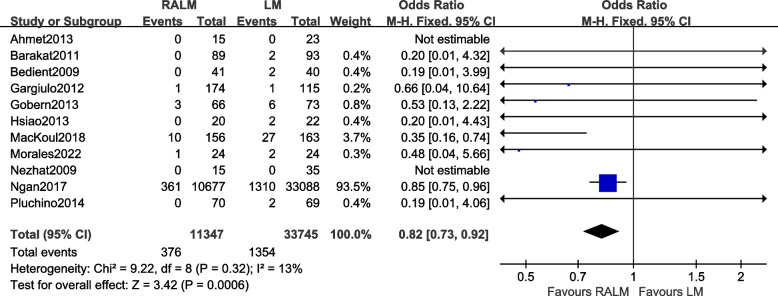
Meta-analysis forest plot of transit open belly rate

A total of 11 studies were included [8–17, 22]. The results [OR = 0.82, 95% CI (0.73, 0.92), *P* = 0.0006] demonstrate a statistically significant difference in the incidence of open bellies between the two surgical approaches. This indicates a lower incidence of open belly in the RALM group compared to the LM group. The meta-analysis results are illustrated in Fig. 6.

#### Comparison of the incidence of postoperative complications

**Fig. 7:**
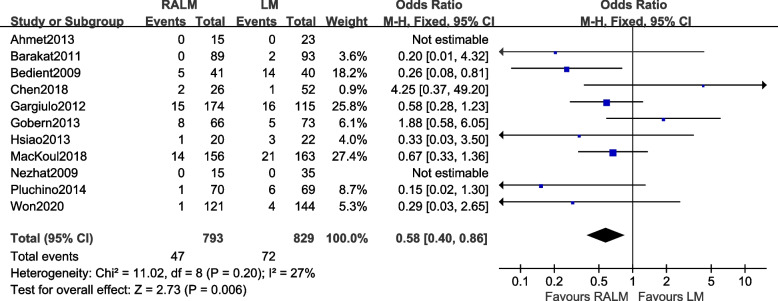
Meta-analysis forest plot of postoperative complication rate

A total of 11 studies were included [8–15, 17, 19, 21]. The results [OR = 0.58, 95%CI (0.40, 0.86), *P* = 0.006] illustrate a statistically significant difference between the two surgical approaches in terms of postoperative complications (endometriosis, postoperative wound infection, bowel injury), indicating that the RALM group has fewer postoperative complications than the LM group. The results of the meta-analysis are shown in Fig. 7.

#### Comparison of the largest myoma diameters

**Fig. 8:**
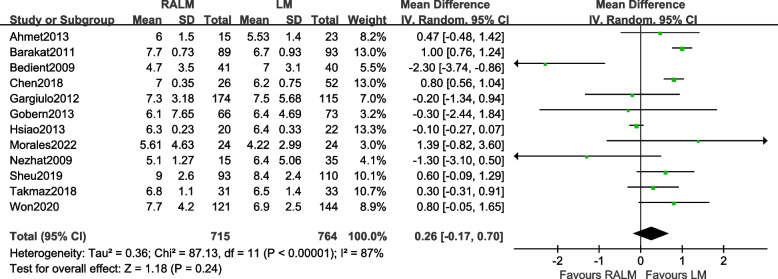
Meta-analysis forest plot of maximum myoma diameter

A total of 12 studies were included [8–14, 18–22]. The results [MD = 0.26, 95%CI (− 0.17, 0.70), *P* = 0.24] indicate that there was no significant difference in maximum myoma diameter between the RALM and LM groups. The meta-analysis findings are presented in Fig. 8.

#### Sensitivity analysis

We sequentially excluded individual studies before combining the analyses for each of the indicators measured, and the results did not change significantly, indicating that the findings of this study are reliable.

#### Publication bias

**Fig. 9:**
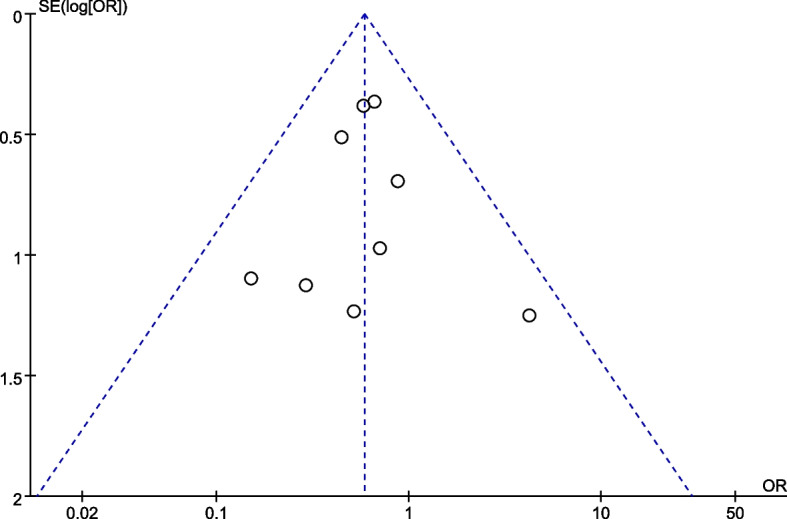
Funnel plot of postoperative complication rate

A funnel plot was drawn with the incidence of postoperative complication as an example, as shown in Fig. 9. It was found that the individual studies were evenly distributed on both sides of the funnel plot and that all studies were distributed inside the funnel plot, indicating that the publication bias of this study was low.

## Discussion

Uterine fibroids are benign tumors formed by the proliferation of smooth muscle tissue in the uterus and are the most common benign tumors in women [23]. Intrinsic abnormalities of the myometrium, abnormal myometrial receptors for estrogen, and hormonal changes or altered responses to ischemic damage during the menstrual period may be responsible for the initiation of (epi) genetic changes found in these tumors [24]. For patients who are not clinically significant, have small fibroids and are not willing to undergo surgery, they can be treated medically or observed at regular follow-up visits. Mifepristone, ulipristal acetate, and hormone analogs are commonly used, but adverse effects limit their long-term use [25]. Surgery is still the main treatment if the fibroids are growing too fast, if malignancy is suspected or if the fibroids are necrotic and conservative treatment has failed. In order to preserve the patient’s fertility as much as possible, clinicians prefer to surgically remove fibroids in patients with indications for surgery, and choosing the best surgical option remains the focus of treatment.

Previous studies [26] have compared the results of laparoscopic and open surgery in the treatment of uterine fibroids and have shown that laparoscopic surgery can reduce intraoperative blood loss, shorten operative time, and shorten postoperative hospital stay. Minimally invasive treatment options also reduce the risk of tissue damage, postoperative pain, and infection, thereby reducing the incidence of postoperative complications [27]. In 2004, Advincula et al. [28] performed the world’s first robotic myomectomy, which laid the foundation for the use of robotic surgical systems in the treatment of uterine fibroids. With the rapid development of minimally invasive surgery, RALM and LM have frequently been chosen by obstetricians and gynecologists. But whether robotic assistance is superior to laparoscopy remains controversial. Therefore, we conducted this meta-analysis to explore and compare the efficacy and safety of RALM versus LM.

The results of this meta-analysis showed that in terms of operative time, the LM group has a shorter operative time than the RALM group. The main reason for this may be due to the inexperience of the gynecologists. RALM was introduced relatively late in some medical centers, and the number of related procedures performed by gynecologists was low, so the surgeons were on an upward learning curve, which may have contributed to the longer procedure times. With the increased experience of gynecologists performing RALM, the procedure time can be comparable to that of laparoscopy. In the USA, gynecologists who are less skilled in the use of laparoscopy may prioritize or prefer the use of robotic surgical systems, as they find the technology easier to learn [29]. It is also important to note that the preoperative preparation of a robotic surgical system is more complex and takes longer to set up than a laparoscopic system, so the actual operative time of a robotic system may be similar to that of a laparoscopic procedure.

In terms of intraoperative bleeding, the results of this study showed that intraoperative bleeding was lower in the RALM group than in the LM group. In the author’s analysis, this is because the robotic surgical system provides a three-dimensional magnified view, greater dexterity, and eliminates hand tremors during surgery, allowing for accurate exposure of the complex anatomy surrounding the resection target. The robotic arm can be rotated 720° and is more flexible than a human hand, allowing finer manipulation than laparoscopy for smoother management of the parametrial vessels [26]. This helps the surgeon to perform precise maneuvers during the procedure and to better control bleeding from small vessels. In terms of the incidence of blood transfusions, the robotic group was lesser than the laparoscopic group. This was the result of less intraoperative bleeding in the robotic group. The study by Montera et al. demonstrated that the use of HEMOPATCH® in laparoscopic hysterectomy achieved hemostatic effects and reduced intraoperative and postoperative bleeding [30]. HEMOPATCH® can be used in both robotic and laparoscopic procedures. Furthermore, the formation of adhesions and the risk of rupture during delivery at the uterine suture site represent supplementary common complications of abdominal myomectomy [30]. In this context, it is important to consider that inadequate hemostasis and the consequent uncontrolled deposition of fibrin are widely believed to contribute to adhesions [31, 32]. Therefore, the reduction in intraoperative and postoperative bleeding due to the hemostatic effect of HEMOPATCH® related to the ability to rapidly and tightly adhere to persistent oozing bleeding, reducing the uncontrolled deposition of fibrin [33]. This may help to reduce the rate of postoperative adhesions by determining the physical barrier between the uterine incision and the adjacent viscera [33].

In terms of postoperative hospital stay, our findings show that patients in the RALM group have shorter postoperative hospital stays than those in the LM group. This is because robotic surgery systems are more minimally invasive. The robotic incision in the abdominal wall consists of a lens hole and three robotic arms and an auxiliary hole, with a total of five 0.5–1.2 cm incisions, resulting in lesser damage than open surgery, more aesthetics, and faster postoperative recovery, resulting in less postoperative pain, faster recovery, and better cosmetic results [26]. Compared with laparoscopy, fine manipulation and more precise control of robotic surgery can reduce the damage to normal tissues during surgery. This also helps to reduce the amount of bleeding during surgery, resulting in shorter postoperative hospitalization and faster recovery time. This also matches well with the concept of enhanced recovery after surgery (ERAS), which is the result of developments in medical theory and surgical techniques that not only place greater emphasis on reducing the patient’s stress response, but also take into account the assessment and intervention of surgical risks [34]. ERAS is a series of optimized measurements for perioperative management to reduce the physical and psychological traumatic stress of surgical patients and achieve rapid recovery [34]. Patients recover more quickly after surgery and also reduce the postoperative hospital stay to some extent. On the other hand, the use of transvaginal specimen retrieval after myomectomy is safe and feasible compared to the removal of the specimen through an incision in the abdominal wall, with the advantages of minimal abdominal scarring and good cosmetic results while ensuring the integrity of the specimen as much as possible, but the impact on sexual life and delivery needs more studies for long-term follow-up [35, 36]. There is currently a lack of studies comparing the risk associated with specimen removal, morcellation, and/or malignant dissemination between robotics and laparoscopy. We hope that future research will further explore this aspect.

The incidence of postoperative complications is an important indicator for assessing short-term postoperative outcomes. The results of this study showed a lower rate of postoperative complications in the RALM group than in the LM group. The robotic surgical system is less invasive to the patient and reduces the risk of bleeding, infection, and adhesions, so the overall complication rate is lower than that of laparoscopic surgery. In addition, in the experience of the author’s center, good cooperation between a gynecologist familiar with the robotic surgical system and an assistant with extensive experience can reduce the incidence of postoperative complications and may influence the outcome of the procedure. The RALM group has a greater advantage when it comes to a mid-turn open belly. However, we need to note that while the RALM group has a lower rate of intermediate openings than the LM group, the robotic surgical system does not facilitate acute openings.

With the maturation of minimally invasive techniques, the use of laparoscopy in gynecological conditions became more widespread. However, because of the lack of a surgical triangle in the laparoscopic system and the limited operative space, suturing and knotting are relatively difficult, making the operator more fatigued [19, 21], and the difficulty of laparoscopic myomectomy increases with the number or size of fibroids; laparoscopic surgery can be difficult for large numbers of fibroids and large fibroids or for fibroids in specific locations [37]. The da Vinci robotic surgical system can effectively overcome these difficulties and provide the technical guarantee for the performance of difficult operations. The da Vinci robotic surgery system consists of a surgeon’s operative table, a mobile robotic arm, and a 3D imaging system [38]. Firstly, the three-dimensional imaging of the robotic system and the magnification of 10 to 15 times gives the operative physician a clearer, three-dimensional view; secondly, the robotic system is equipped with EndoWrist laparoscopic instruments with a range of motion of 7 degrees of freedom, allowing for greater flexibility in the abdominal cavity; thirdly, the robotic system filters out hand tremors, making the operation more stable and safe; finally, the operator only needs to sit in front of the operative table to operate the robot system, which greatly reduces the operator’s physical effort, and the operator operates in a relatively comfortable position to reduce the occurrence of intraoperative errors [38].

Limitations of this meta-analysis: (i) there was significant heterogeneity in operative times, and potential factors for this heterogeneity included a differential experience of gynecologists and a shorter learning curve in the robotic group; (ii) the included studies are retrospective clinical studies where selection bias is inevitable and needs to be validated by a larger sample of randomized controlled trials; (iii) only literature in Chinese and English was included; there may have been literature in other languages that met our inclusion criteria but were not included due to language restrictions; and (iv) the small sample size of some of the included studies may have caused bias in the analysis. Despite these limitations, our study provides new insights into the efficacy and safety of RALM versus LM.

## Conclusion

In summary, by meta-analysis of the included literature, we found that LM has a shorter operative time, while RALM has an advantage over LM in terms of intraoperative bleeding, number of blood transfusions required, length of hospital stay, rate of intermediate openings, and postoperative complications, suggesting that RALM is superior to LM in terms of surgical trauma and postoperative recovery. However, there is a lack of long-term postoperative follow-up studies of patients and we look forward to the publication of larger sample, high-quality randomized controlled studies in the future.

## Data Availability

The datasets used and/or analyzed during the current study are available from the corresponding author on reasonable request.

## Abbreviations

RALM: Robotic-assisted laparoscopic myomectomy
LM: Laparoscopic myomectomy
BMI: Body mass index
OR: Odds ratio
MD: Mean difference
CI: Confidence interval
ERAS: Enhanced recovery after surgery
